# Shared endo-phenotypes of default mode dysfunction in attention deficit/hyperactivity disorder and autism spectrum disorder

**DOI:** 10.1038/s41398-018-0179-6

**Published:** 2018-07-17

**Authors:** Julius M. Kernbach, Theodore D. Satterthwaite, Danielle S. Bassett, Jonathan Smallwood, Daniel Margulies, Sarah Krall, Philip Shaw, Gaël Varoquaux, Bertrand Thirion, Kerstin Konrad, Danilo Bzdok

**Affiliations:** 1 Department of Psychiatry, Psychotherapy and Psychosomatics, RWTH Aachen University, 52072 Aachen, Germany; 2 Department of Psychiatry, University of Pennsylvania, Perelman School of Medicine, Philadelphia, PA 19104 USA; 3 Department of Bioengineering, University of Pennsylvania, Philadelphia, PA 19104 USA; 4 Department of Electrical & Systems Engineering, University of Pennsylvania, Philadelphia, PA 19104 USA; 5 Department of Psychology, York Neuroimaging Centre, University of York, Hesslington, York, UK; 6 Max Planck Institute for Human Cognitive and Brain Sciences, 04303 Leipzig, Germany; 7 Child Psychiatry Branch, National Institute of Mental Health, Bethesda, MD 20892 USA; 8 Parietal team, INRIA, Neurospin, bat 145, CEA Saclay, 91191 Gif-sur-Yvette, France; 9 JARA-BRAIN, Jülich-Aachen Research Alliance, Aachen, Germany; 10 Department of Child Psychiatry, Child Neuropsychology Section, RWTH Aachen University, 52072 Aachen, Germany; 11 Institute of Neuroscience and Medicine (INM-3), Research Centre Juelich, Juelich, Germany

## Abstract

Categorical diagnoses from the Diagnostic and Statistical Manual of Mental Disorders (DSM) or International Classification of Diseases (ICD) manuals are increasingly found to be incongruent with emerging neuroscientific evidence that points towards shared neurobiological dysfunction underlying attention deficit/hyperactivity disorder and autism spectrum disorder. Using resting-state functional magnetic resonance imaging data, functional connectivity of the default mode network, the dorsal attention and salience network was studied in 1305 typically developing and diagnosed participants. A transdiagnostic hierarchical Bayesian modeling framework combining *Indian Buffet Processes* and *Latent Dirichlet Allocation* was proposed to address the urgent need for objective brain-derived measures that can acknowledge shared brain network dysfunction in both disorders. We identified three main variation factors characterized by distinct coupling patterns of the temporoparietal cortices in the default mode network with the dorsal attention and salience network. The brain-derived factors were demonstrated to effectively capture the underlying neural dysfunction shared in both disorders more accurately, and to enable more reliable diagnoses of neurobiological dysfunction. The brain-derived phenotypes alone allowed for a classification accuracy reflecting an underlying neuropathology of 67.33% (+/−3.07) in new individuals, which significantly outperformed the 46.73% (+/−3.97) accuracy of categorical diagnoses. Our results provide initial evidence that shared neural dysfunction in ADHD and ASD can be derived from conventional brain recordings in a data-led fashion. Our work is encouraging to pursue a translational endeavor to find and further study brain-derived phenotypes, which could potentially be used to improve clinical decision-making and optimize treatment in the future.

## Introduction

Attention deficit/hyperactivity disorder (ADHD) and autism spectrum disorder (ASD) are both disabling and heritable neurodevelopmental disorders that manifest early in life and have well-documented consequences for well-being. Both disorders are associated with high levels of family dysfunction, social interaction problems, academic failure, and unemployment and thus constitute a significant burden for children, their families, and society as a whole^1–3^.

ADHD is characterized by developmentally inappropriate levels of inattention, impulsivity, and hyperactivity. In contrast, ASD is defined by core symptoms of persistent and pervasive deficits in social communication and interaction along with repetitive behavioral patterns and restricted interests or activities. However, these seemingly disparate disorders have clinical overlap^4^: 30–80% of all ASD children meet the diagnostic criteria for ADHD and, conversely, 20–50% of children diagnosed with ADHD also meet the diagnostic criteria for ASD. Both disorders also show similar associated clinical features, including poor social skills, language delay, oppositional defiant behavior, and difficulty with attention and emotion regulation^4,5^. This begs the question whether despite superficial differences in clinical presentation both ADHD and ASD share a fundamental mechanism of dysfunction.

Consistent with the hypothesis that both ASD and ADHD depend in part on shared underlying dysfunction, genetic and twin studies show familial associations for both disorders^6,7^. Twin studies suggested that 50–72% of phenotypic features are shared by these disorders, potentially reflecting genetic factors common to both ADHD and ASD^8,9^. Additionally, genome-wide association studies as well as linkage and candidate gene studies identified a number of genetic risk variants common to both disorders^10^. At the neuropsychological level, there are several domains in which both ASD and ADHD have a pattern of common deficits. These include executive function^11^, emotion recognition^12^, affective feedback processing^13^, as well as sustained attention, and sensory functioning^14,15^.

Independent functional magnetic resonance imaging (fMRI) experiments in ADHD or ASD patients have revealed a substantial role of aberrant connectivity in large-scale networks in both disorders (for reviews see refs.^16,17^). Prior evidence has emphasized the importance of the default mode network (DMN) and attention-related macroscopical network as a key to both ADHD and ASD dysfunction^18–20^. In a seminal cross-diagnostic neuroimaging study, Di Martino et al.^20^ examined network centrality metrics in ADHD and ASD patients. Abnormalities were identified in cortical and subcortical areas, some of which were common to both disorders, including the posteromedial cortex. In contrast, some aberrations, such as limbic areas in the bilateral medial temporal lobe, were more closely related to ASD. Moreover, it has been suggested that the salience network (SN) is intimately related to the interplay between the DMN and DAN^21^, and aberrant coupling patterns between the SN, DMN, and DAN have been reported in both ASD^18,22^ and ADHD^23,24^.

The collection of genetic, neuropsychological, and neuroimaging evidence emphasizes the need to understand the common patterns of neural dysfunction that link ADHD and ASD. Both disorders may be best understood from a dimensional point of view with patients who suffer from either disorder located at distant points on a symptom continuum^8^. This intuition is advertised by the Research Domain Criteria (RDoC) initiative of the National Institute of Mental Health^25^ proposed as an alternative research framework to investigate psychopathological disorders, including ADHD and ASD. Within this framework, mixed dimensional abnormalities of brain circuits are conceptualized as an underlying dysfunction that can contribute to clinically diverging mental disorders to varying degrees^26,27^. In the present study, we tested a dimensional view of ADHD and ASD combining resting-state brain connectivity and emerging tools from the machine learning domain. In a transdiagnostic fashion, we hypothesized that brain variation in large-scale network connectivity in the DMN, DAN, and SN can be used to identify shared fundamental network dysfunction in both disorders.

## Methods

### Data resources and preprocessing

Already preprocessed neuroimaging data were obtained from two large, publicly available datasets: ADHD-200 (http://fcon_1000.projects.nitrc.org/indi/adhd200/) and ABIDE (Autism Brain Imaging Data Exchange; http://fcon_1000.projects.nitrc.org/indi/abide/). All data were anonymized, and collected with the approval of the respective ethics boards. Experienced psychiatrists performed patient diagnoses. The ADHD-200 data set provides demographic and clinical information, including age, sex, and measures of symptom severity as assessed by the ADHD rating scale (ADHD-RS). The ABIDE data provide subject information, including age, sex, and measures of symptom severity as assessed by the Autism Diagnostic Observation Schedule (ADOS). Both considered data repositories were preprocessed using the NeuroImaging Analysis Kit (NIAK, http://preprocessed-connectomes-project.org, for in-depth description see refs.^28,29^). Particular care has been devoted to help mitigate motion artefacts: Scrubbing^30^ was used to remove volumes with excessive motion. Rigid-body motion was then estimated within and between runs. The first principal component accounting for 95% of the variance of the six rigid-body motion parameters, as well as their squares was regressed out in nuisance removal. The available pipeline was additionally modified using a standard removal of linear effects with site as a regressor of no interest to control for certain acquisition-related effects.

To help minimize confounding factors, inclusion was restricted to children and adolescents who were male and between 7 to 21 years of age to study the neural mechanism of both disorders during development. Diagnosed and typically developing (TD) participants were age-matched in each dataset (see Table 1 for details). This was motivated by previous evidence showing that ASD affects the brains of children and adults differently^31^. Further, we included only male participants because (i) both disorders are more prevalent in males^32,33^, and (ii) to exclude gender-specific differences in brain heterogeneity^34,35^. Based on these selection criteria, 587 age-matched participants (303 TD) from ADHD-200, and 718 age-matched participants (349 TD) from the ABIDE repository were eligible. This amounted to a total of *n* *=* 1305 participants.

**Table 1:** Sample details

ADHD-200 (*n*=587)	ADHD	TD	*p*-value (*t*-test)	ABIDE (*n*=718)	ASD	TD	*p*-value (*t*-test)
*n*	284	303		*n*	369	349	
Age	11.99	11.89	>0.99	age	13.53	13,54	>0.99
*ADHD subtypes*				*ASD subtypes*			
Inattentive (%)	35.00	0.00		Autism (%)	75.00	0,00	
Hyperactive/ Impulsive (%)	4.00	0.00		Asperger (%)	18.00	0,00	
Combined (&)	61.00	0,.0		PDD-NOS (%)	7.00	0,00	
ADHD symptom severity	62.00	38.00	<0.001	ADOS total	12.00		
Inattention	55.00	33.00	<0.001	ADOS communication	4.00		
Hyperactivity/Impulsivity	52.00	32.00	<0.001	ADOS social interaction	8.00		
				ADOS stereotyped behaviors	3.00		

### Target network definition

**Fig. 1:**
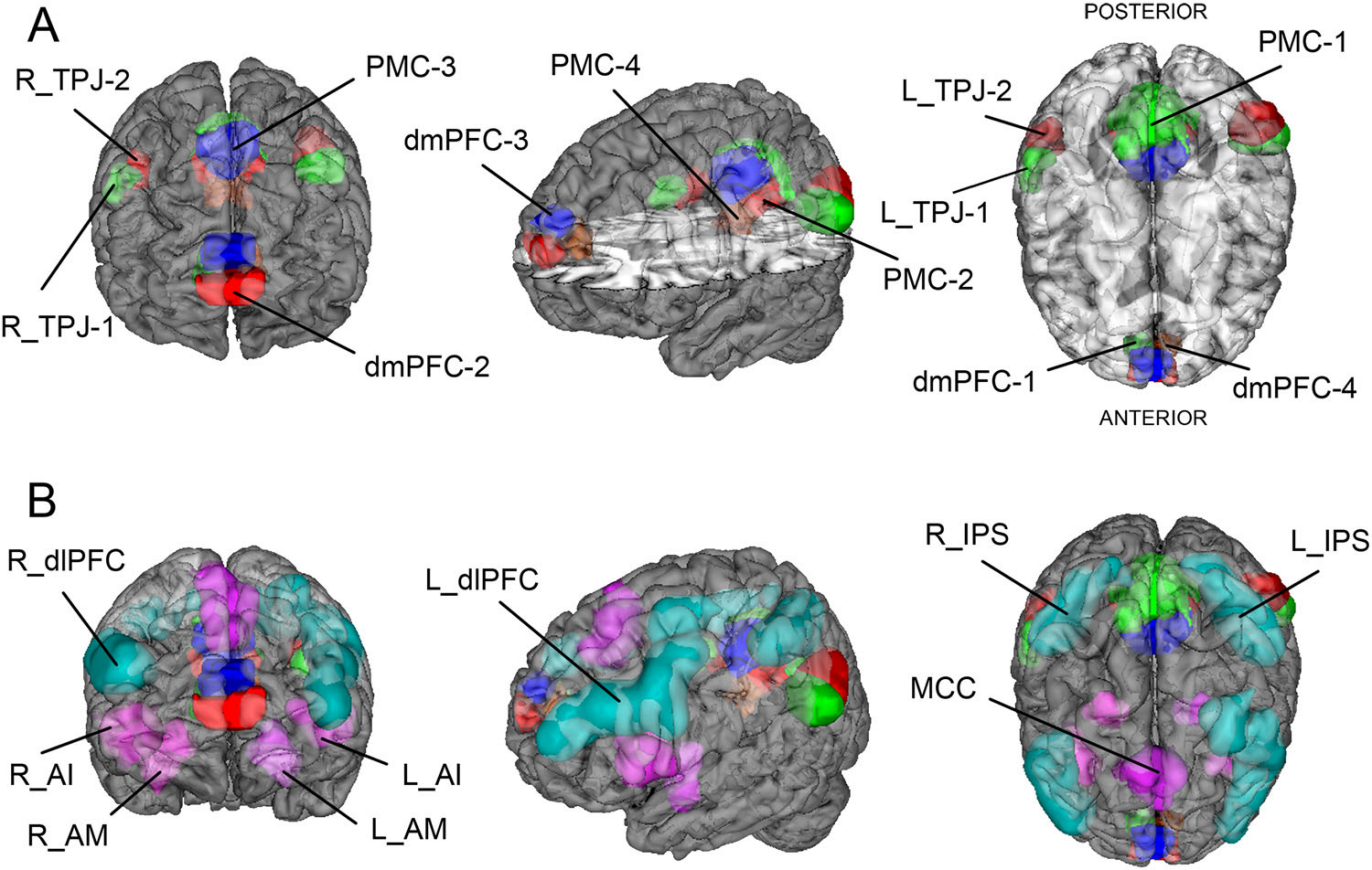
Target network definitions. The regions of interest (ROIs) used for all present analyses are rendered on the MNI standard brain with frontal, diagonal, and top views. **a** The four main default mode network (DMN) nodes are subdivided into 12 ROIs reflecting distinct subregions (dmPFC1–4, PMC1–4, left and right TPJ1–2)^37–40^. **b** The DMN subregions are supplemented by nine ROIs for the dorsal attention network (DAN) and salience network (SN), drawn from previously published quantitative meta-analyses. The DAN was composed of the dorsolateral prefrontal cortex (dlPFC) and intraparietal sulcus (IPS) bilaterally^42^. The SN included the anterior insula (AI), midcingulate cortex (MCC), and amygdala (AM) bilaterally^41^. NeuroVault permanent link to all ROI definitions used in the present study: http://neurovault.org/collections/2216/

For each participant, the preprocessed resting-state connectivity was summarized in network-coupling statistics. We examined several subregions *within* each of the four DMN nodes (Fig. 1a) as used in a recent computational psychiatry study (see ref.^[36^; available for re-use at http://neurovault.org/collections/2216/): four subregions in the dorsomedial prefrontal cortex (dmPFC), four subregions in the posteromedial cingulate cortex (PMC), and two subregions in the right and left temporoparietal junction (TPJ) were drawn from a recently completed quantitative meta-analytical atlas of the DMN derived by connectivity-based parcellation^37–40^. The DMN nodes were supplemented by coordinate-based meta-analyses of closely interacting multi-modal networks (Fig. 1b): the salience network, composed of the anterior insula (AI), midcingulate cortex (MCC), and amygdala (AM)^41^; and the dorsal attention network (DAN), composed of the dorsolateral prefrontal cortex (dlPFC) and intraparietal sulcus (IPS)^42^. This approach yielded a total of 21 nodes with 210 edges capturing functional network coupling between all possible connectivity pairs. The fMRI signal was summarized by an average time-series for each node, standardized by zero-meaning and unit-variance scaling, and detrended. Pearson’s correlations were then computed between each possible pair of the network nodes. In this way, we effectively reduced each individual’s resting-state whole-brain information to an interpretable set of connectivity variables. In sum, the set of coupling measures reflects each subject’s specific connectivity profile—analogous to a fingerprint of brain network connectivity. Constructing analogous connectivity variables from networks in the Yeo atlas^43^—without DMN, DAN, and SN—yielded only 52.65% accuracy in the autism-health distinction and 56.06% accuracy in the ADHD sample (100 cross-validation folds, 90% train, and 10% test set, linear support vector machine (SVM)).

### Statistical analysis

**Fig. 2:**
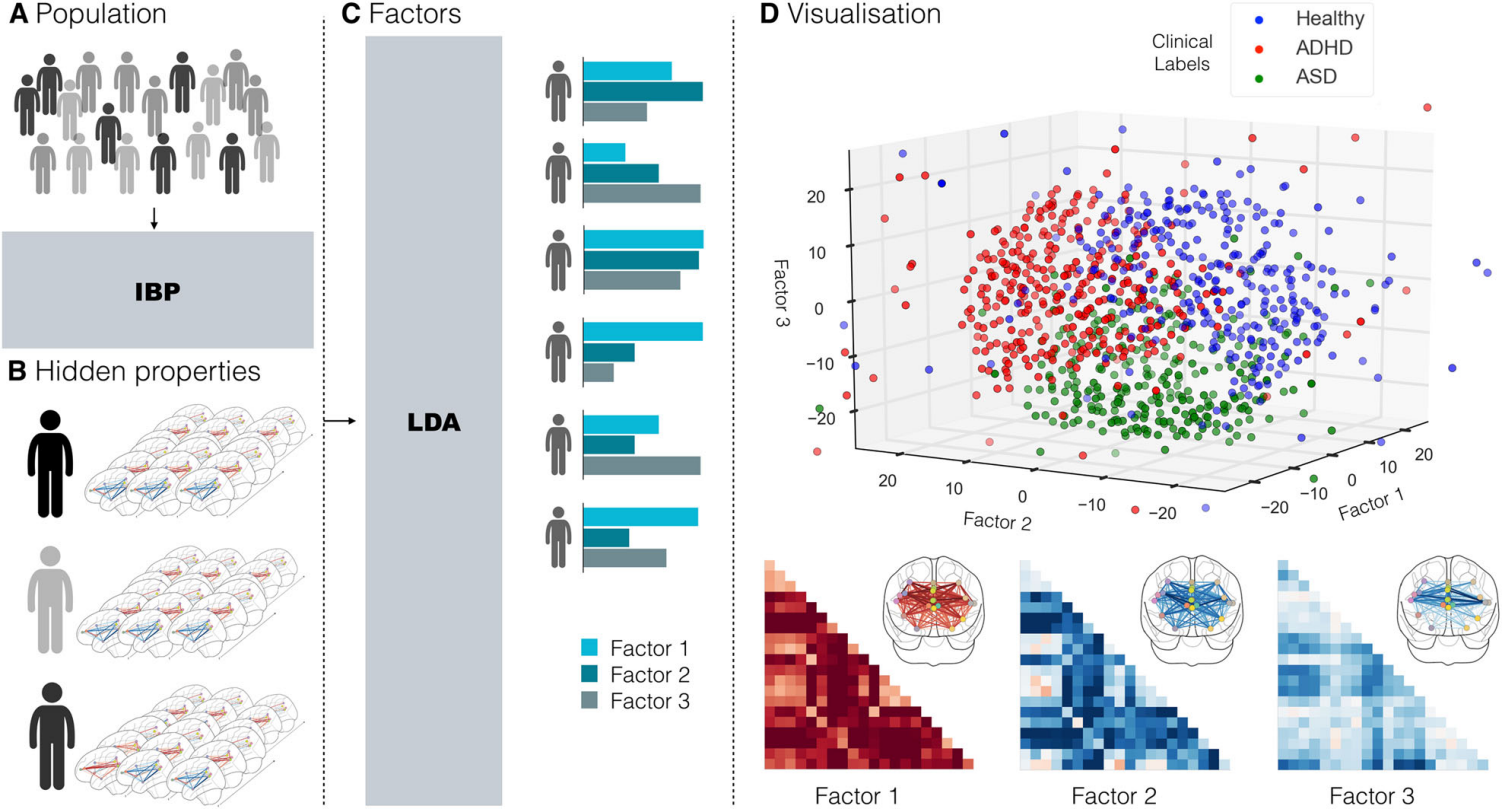
Workflow. **a** DMN, DAN, an SN network coupling was studied in a composite sample of 1,305 TD, ADHD, and ASD individuals taken from two multisite open-data repositories (ADHD-200 and ABIDE). **b** In a data-driven fashion, *Indian Buffet Processes* (IBP) automatically derived the number of hidden properties in the connectional fingerprints across participants without recourse to their clinical status. Automatic detection and weighing of shared and distinct unknown biological causes prompts its use in the identification of endo-phenotypes. **c** *Latent Dirichlet Allocation* (LDA) then inferred three overarching factors of underlying brain variation. Importantly, LDA allowed to derive hidden variability factors with mixed membership. Therefore, each participant’s connectional fingerprint was modeled to be simultaneously caused by multiple implicit neurobiological factors. **d** Each individual composition of the three neurobiological factors (representing distinct network-coupling profiles, *lower section*) was related to their respective clinical diagnoses (TD, ADHD, and ASD). In a preliminary analysis based on t-distributed stochastic neighbor embedding (t-SNE; ref.^62^), biological subtypes can be identified from network connectivity patterns that are partly shared across TD, ADHD, and ASD

In this study, we devised an innovative hierarchical Bayesian modeling strategy (Fig. 2) to address the urgent need for objective brain-derived measures that can acknowledge shared dysfunction leading to different brain disturbances across disorders, including ADHD and ASD. The applied transdiagnostic framework is able to reflect the premise that different underlying pathophysiological mechanisms contribute to mental disorders to varying degrees^26,27^. In the following, we will now describe step-by-step what key advantages the applied framework offers.

### Identification of underlying disease dimension

In a first step, we wanted to identify the hidden components of disease variability underlying the connectivity profiles. The challenges implicated are to do so in a data-led fashion, imposing minimal constraints (such as selecting a pre-specified number of components), and to allow for the contribution of multiple shared components at the same time. In an early application in neuroimaging, we used *Indian Buffet Processes* (IBP)^44^ to allow for the derivation of the *relative* contributions of hidden properties in the connectivity profiles across all participants. Rather than extracting a pre-specified number of components, as commonly used in principal or independent component analysis, IBP enables formal inference on the *number* of unknown components. This non-parametric model hence automatically determines the number of underlying components flexibly adapted to the richness of the available directional functional-connectivity data. Additionally, IBP does not perform *hard assignments*; instead it associates hidden properties to patterns of continuous variation in particular node–node couplings rather than to binary differences.

### Hierarchical Bayesian modeling

The identified hidden properties in functional network coupling then provided the basis for drawing inference of coherent group-overarching structure (i.e., factors) by means of Bayesian hierarchical modeling. Using *Latent Dirichlet Allocation* (LDA)^45^ we imposed a hierarchy of pre-specified *k* number of factors onto the connectivity fingerprints based on their association with the hidden properties. In previous research, LDA was successfully applied after engineering structural brain data into positive integers^46^. But LDA alone is not suited to handling *negative-valued, non-discrete* input, such as connectivity strengths. Here, the realized combination of IBP and LDA modeling naturally suggests itself because IBP can seamlessly transform the *continuous* information encoded in the individual connectional fingerprints into *discrete*, *positive-valued* vectors indicating the assignment to the underlying hidden properties. For ease of interpretation, LDA then reduced the obtained set of assignments to hidden properties into a small set of overarching connectivity archetypes (i.e., factors). A key advantage of combining IBP and LDA is that it enables us to derive hidden sources of variation with mixed memberships. This avoids the necessity of assigning a connectional fingerprint of a participant to only one factor. Instead, each particular individual’s connectional fingerprint could hence be modeled as being generated by *k* factors (i.e., endo-phenotypes) *simultaneously.*

### Deriving biological labels from the neuroimaging-derived phenotypes

We generated an unbiased set of new labels indicating an assignment to a ‘neurobiological group’ based on the dimensional factors constituting the brain phenotypes for all individuals. To avoid circularity, we translated a statistical modeling scheme, called *pre-validation*^47^, to the neuroimaging domain. As a variant of cross-validation, pre-validation was applied to obtain a fairer evaluation of the group labels^48^. While cross-validation yields reasonably unbiased estimates of the model’s expected error rate in other observations, pre-validation produces a new set of unbiased data or labels that mimic the model performance in later recruited subjects labeled as patients and controls^49^. These authors emphasize that the key feature of pre-validation is that each label is derived from the entire data set and independently of its response value. Therefore, each label can be treated as if it was derived from a data set completely separate from the test-data. The biological group labels hence are statistically independent from the information encoded in the connectional fingerprints^48–51^, and act as if they were derived from separate data.

We divided the data into *m* = 10 pseudo-randomized splits to ensure balanced groups in both training and test set. The biological labels for all individuals in a given *m*-th data split were generated by fitting an LDA model on combined brain data from the nine remaining data splits and used to infer factor weights for all observations of the *m*-th data split. In each *m*-th data split, pre-validated biological labels were hence estimated by LDA (i.e., the “internal model”) without access to any actual clinical labels (TD versus ADHD versus ASD) or any brain data from the held-out *m*-th data split. This procedure generated a new set of labels that was then used to evaluate the out-of-sample prediction of the groups based on a linear classification algorithm (whereas classical cross-validation directly selects models and evaluates their prediction performance). The biological labels were tested for diagnostic relevance based on linear SVMs (i.e., the “external model”) by training on each combination of *m*−1 training data splits and testing on the respective remaining test-data split.

## Results

A hierarchical Bayesian approach was used to identify distinct patterns of DMN coupling with other large-scale brain networks. These functional network patterns were consistently expressed in each of the 1305 TD, ADHD, and ASD individuals from two multisite repositories (i.e., ADHD-200 and ABIDE). The applied transdiagnostic modeling strategy reflects the premise that different biological phenotypes contribute to clinically diverging mental disorders to varying degrees^26,27^. After automatic extraction of distinct variability components in DMN coupling (i.e., hidden properties), we inferred a hierarchy of sources of variation (i.e., factors) that compile the variability in network connectivity of the DMN in TD and diagnosed participants.

**Fig. 3:**
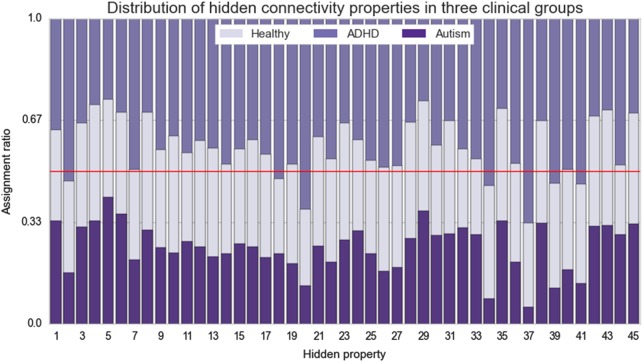
Hidden properties in connectivity profiles. Healthy (middle section in the columns), ADHD (upper section in the columns), and ASD (lower section in the columns) participants are compared with regard to the relative occurrence of each distinct hidden component (columns). Each hidden property resulted directly from the Indian Buffet Process and is depicted here with its occurrence (present versus not present) added up across all participants. These were automatically discovered in the whole-brain connectivity profiles without knowing to which of the three groups each participant belonged. Visibly, the identified connectivity features are dispersed across the participant groups. No single connectivity feature was exclusively associated with only one group

The hidden properties of disease variability underlying the connectivity profiles were identified in a data-driven fashion across all participants without knowing to which clinical group (TD, ADHD, or ASD) they belonged to. The applied non-parametric model automatically determined *45 hidden properties* as the *number of components* adapted to the complexity of the underlying the available data. We then investigate whether distinct disorder-specific clusters would emerge. However, while every hidden property was observed to be present to different extents in each diagnostic group, no property was found to be uniquely associated with only one group (Fig. 3). Together this provides initial evidence that different biological phenotypes are partly shared among individuals and contribute to the clinical presentation of ADHD and ASD to varying degrees.

**Fig. 4:**
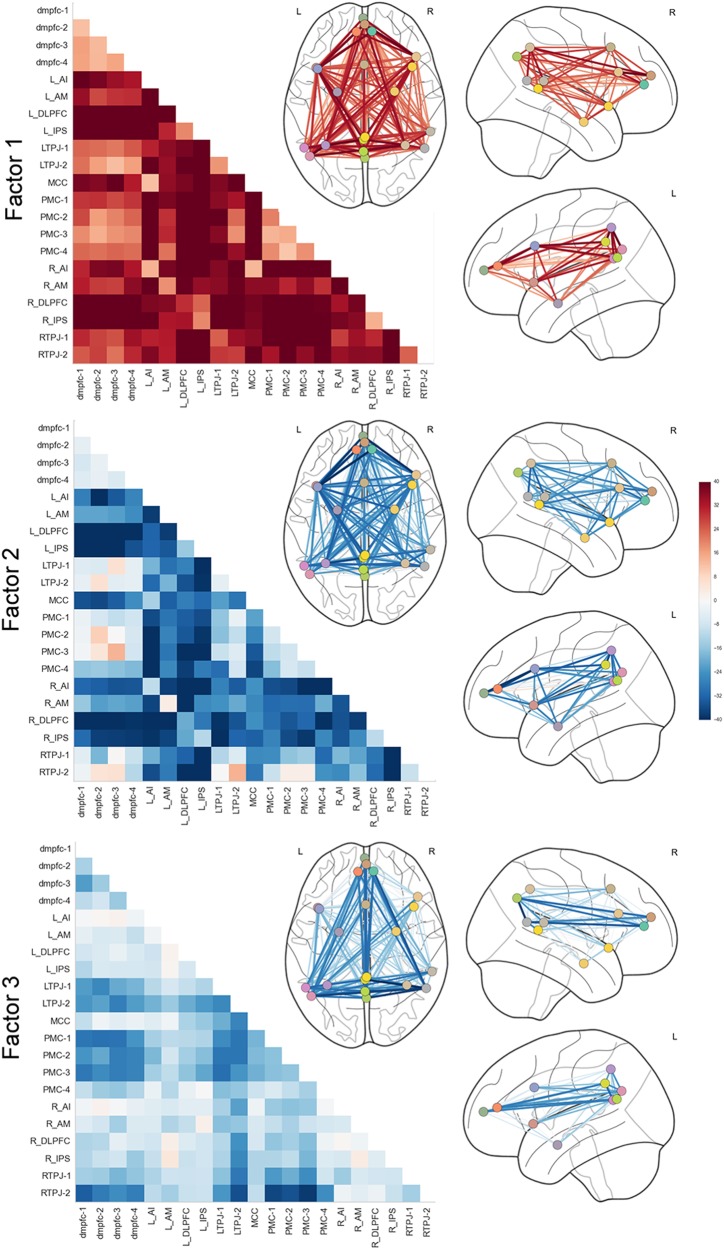
Three neurobiological factors of variation with distinct connectivity patterns. Bayesian inference allowed extracting a hierarchy of brain-defined subgroups, without access to the clinical diagnoses. Each of the three biological factors reflected a coherent pattern of resting-state connectivity between the default mode network (dmPFC-1/2/3/4, PMC-1/2/3/4, and bilateral TPJ-1/2), dorsal attention network (bilateral dlPFC and IPS), and salience network (bilateral AI, MCC, and AM). In each TD, ADHD, or ASD individual, the resting-state measurements of overall network-coupling patterns were driven by flexible recombinations of these three factors of connectivity variation. L/R left/right hemisphere

To aid interpretability, we then used Bayesian inference to reduce the obtained set of hidden properties into a smaller set of overarching patterns by imposing a latent hierarchy of *k* factors. In the *k* = 2 solution, the underlying factors were only related in opposite directions and were hence not able to capture subtle effects in overall network coupling. In wanting to choose the lowest yet most informative number of hidden factors, we favored a solution with *k* = 3 factors. Hypothetically, if the three clinical groups were to be neurobiologically consistent, three learned LDA components would suffice to describe the underlying dysfunctional pattern. For instance, LDA factor 1 could be related to healthy subjects, LDA factor 2 to ADHD, and factor 3 to ASD. However, following the shared hidden properties, we found that the three factors did not align in a one-on-one fashion with the clinical groups (cf. Fig. 1d). Consistent with our hypothesis, the shared influence of three connectivity factors was associated with aspects of both ASD and ADHD. The identified factors yielded the following coupling weights (Fig. 4): Factor 1 showed high DMN-DAN, medium DMN-SN, and low intra-DMN coupling weights, while factor 2 exhibited positive weights for connections between DMN subregions, most pronounced for the right and left posterior TPJ, and between the right and left AM. The highest negative weights of factor 2 were observed for connections between the dmPFC subregions and the right and left dlPFC, closely followed by the right and left IPS. Factor 3 exhibited subtle effects for connections between DMN subregions. The connections between the right posterior TPJ and the PMC, and between the right and left posterior TPJs showed particularly high negative weights. In sum, each of the biological three factors reflected a coherent pattern of resting-state connectivity between the DMN, DAN, and SN. Capitalizing on the mixed memberships approach of our framework, each individual’s resting-state network connectivity could hence be expressed as a flexible *recombination* of only these three factors.

### Clinical associations of the biological phenotypes

We then examined the subject-by-subject expression of the imaging-derived endo-phenotypes (i.e., factors 1–3) in regard to the clinical questionnaires and assessments available from the ADHD-200 and ABIDE repositories. The subject-by-subject expression of factor 1 showed the highest positive associations with ADHD symptom measures, including the level of inattention (*r* = 0.26, *p* < 0.001) and hyperactivity/impulsivity (*r* = 0.24, *p* < 0.001), as well as a negative association with performance, verbal, and total IQ scores (*r* = −0.13/−0.15/−0.13, each *p* < 0.05). In contrast, factor 2 showed the highest associations with ASD diagnosis (*r* = 0.15, *p* < 0.05), and positive associations with verbal and total IQ (*r* = 0.21/0.14, *p* < 0.001/0.05), as well as negative associations with ADHD diagnosis (*r* = −0.22, *p* < 0.001) and hyperactivity/impulsivity (*r* = −0.21, *p* < 0.001). Factor 3 did not show significant associations with any behavioral items.

### Validating the predictive nature of the biological phenotypes against clinical diagnoses

**Fig. 5:**
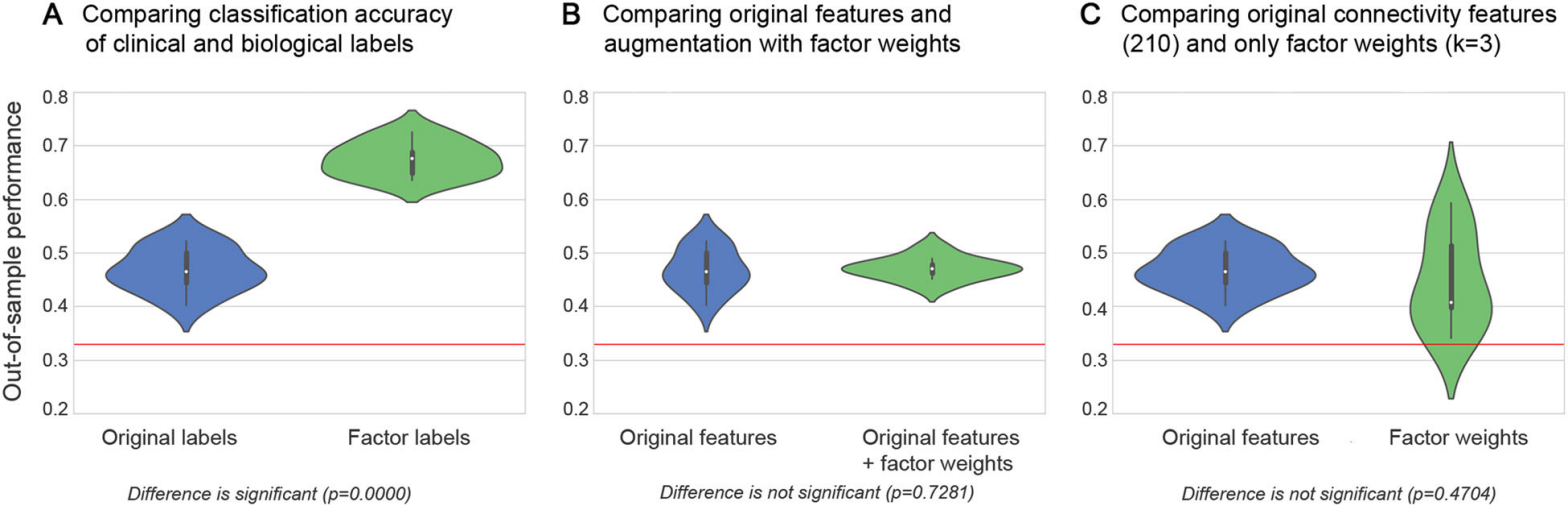
Evaluation of predictability, robustness, and expressiveness of the transdiagnostic brain phenotypes for clinical validation. Evaluating intra-subject predictions, the clinical usefulness of the measured network connectivity strengths (blue) was systematically evaluated against the discovered neurobiological endo-phenotypes (green). Violin plots are similar to box plots in showing the median (white point), quartiles (thick black lines), and outliers (below/above thin black whiskers), but also expose the probability densities of the data points (sideways shapes). **a** Classification performance (1.0 = all subjects correct, 0.33 = chance as red line) of predicting the original diagnosis groups (TD, ADHD, and ASD) versus the neurobiologically derived groups (indicated by the most important factor in each participant) based on the overall brain connectivity. The data-derived disease factors could be much better predicted in connectivity profiles from new, previously unseen participants (*p* < 0.0001). **b** Classification performance of predicting the original diagnosis groups based on connectivity profiles versus connectivity profiles and additional factor weights. Knowledge of the brain-derived disease factors much decreased the variance (concentration around medium), thus decreasing the uncertainty of each prediction for a given participant. **c** Group prediction performance from full connectivity profile versus exclusive knowledge of the brain-derived factor weights. Without direct access to the original brain connectivity measurements, three factor weights summarizing each subject were sufficient for non-inferior prediction (*p* = 0.47). The brain-imaging-derived phenotypes hence improved predictability, robustness, and expressiveness

In a final step, we explored the association between the discovered brain-derived connectivity factors and the biological and categorical labels (Fig. 5). Note that the connectivity factors and biological labels were derived without using the original disease group labels or any questionnaire scores. To enable systematic assessment of the predictive accuracy added by the discovered dimensional endo-phenotypes, we generated an unbiased set of new data-derived neurobiological labels for all individuals. The neurobiological labels were then systematically compared against the clinical labels by testing for diagnostic relevance based on linear SVMs. We conducted three plausibility tests to provide quantitative answers to different questions.

(1) We asked whether the new data-derived neurobiological labels capture the neural dysfunction encoded in the connectional fingerprints more accurately than the categorical labels (i.e., TD versus ADHD versus ASD) (Fig. 5a). We would like to point out that all biological labels were statistically independent of the connectivity fingerprint and therefore act just like a regular input variable (c.f. pre-validation in methods)^50,51^. SVMs correctly predicted the independent neurobiological label from connectional fingerprints in unseen participants 67.33 ± 3.07% of the time (chance is at 33.33%). Predicting the original categorical diagnoses provided by board-certified psychiatrists achieved only an accuracy of 46.73 ± 3.97% in new participants. This difference in classification accuracy across predictions was statistically significant at *p* < 0.0001 as evaluated by a *t*-test. This finding indicates that the imaging-derived neurobiological labels captured the underlying variation of disease dimension within the connectivity information more accurately than the original categorical group labels.

(2) We explored whether the categorical diagnostic labels could be better predicted from the individual connectional fingerprint (i.e., the full node–node connectivity information for each participant) if the factor weights were added to the explanatory variables (Fig. 5b). We hence asked whether adding the information about the individual factor weights (i.e., three continuous numbers) to the connectional fingerprint enhances the diagnostic classification to capture the underlying shared pathology more accurately. The classification accuracy on the original connectivity fingerprints alone reached 46.73 ± 3.97 percent (chance still at 33.33%), whereas the original features supplemented with the weights of biological factors reached 46.61 ± 1.98%. When adding the dimensional information of the biological groups, there was hence no statistically significant difference in out-of-sample prediction accuracy (*p* = 0.73). However, notably, the prediction model improved according to another clinically relevant performance metric: The variance of the prediction model was reduced by a factor of 2. This finding indicated that aiding the prediction model based on categorical group labels by adding information on the biological groups did not enhance categorizing the shared neuropathology reflected in the sets of connectivity features on average across predictions, but made prediction in a given individual more reliable.

(3) We compared the predictability of the categorical labels based on the full connectional fingerprint with the predictability based on the three factor weights alone (i.e., a total of 3 numbers per participant; Fig. 5c). The analysis achieved a classification performance of 44.48 ± 9.11% accuracy in unseen participants based on the factors, and was very close to the 46.73 ± 3.97% accuracy in prediction of the clinical labels based on the full connectivity matrix. This difference in prediction performance was not statistically significant (*p* = 0.47). To emphasize the importance of this finding: Reducing the 210 node–node connectivity features to three indicators of biological phenotypes in each individual still allowed for classification of TD, ADHD, and autistic participants with essentially identical predictive performance.

In summary, we identified imaging-derived brain phenotypes based on large-scale network connectivity in the DMN, DAN, and SN using a hierarchical Bayesian framework. The phenotypes were derived in a data-driven fashion without access to any clinical or diagnostic information, and were gradually shared across TD, ADHD, and ASD individuals. Finally, we demonstrated that these brain endo-phenotypes were reliable to enhance categorical diagnoses made by board-certified psychiatrists to capture the underlying neural dysfunction shared in both disorders more effectively.

## Discussion

The present computational investigation sought formal models to capture the shared neural dysfunction in ADHD and ASD. Given the overlap in clinical presentation (i.e., exo-phenotypes), we hypothesized that distinct neural signatures (i.e., endo-phenotypes) can be found to describe the common underlying brain network dysfunction. We introduced a novel framework of hierarchical Bayesian inference to identify brain phenotypes of DMN coupling, which were gradually shared across 1305 TD, ADHD, and ASD individuals. We showed that both disorders could be situated along three dimensions of neurobiological variation. We decided to focus our study on previous empirical evidence for shared abnormal large-scale network function in ADHD and ASD. The present data hence suggest that the clinical overlap seen in ADHD and ASD is caused by a shared underlying pattern of brain network dysfunction characterized by distinct coupling patterns of the temporoparietal cortices in the DMN with the DAN and SN. In the following, we discuss the coupling patterns of each factor in the light of the current neuroimaging literature.

Factor 1 was characterized by high DMN-DAN, medium DMN-SN, low intra-DMN, and low intra-DAN coupling weights. The subject-by-subject expression of this factor showed the highest positive associations with ADHD symptom measures. These observations largely confirm previous findings that the manifestation of ADHD symptoms involves altered DMN-DAN interactions, e.g. as implicated in attentional lapses^52^. Our results are consistent with reports of decreased connectivity within the DMN and DAN in ADHD populations^19,23^, which the investigators proposed to explain attention deficits. In contrast to the behavioral associations of factor 1, the subject-specific expression of factor 2 was positively correlated with ASD diagnosis. On a network level, factor 2 showed high negative functional connectivity for DMN-DAN, low DMN-SN and AI-AM connections. This confirmed and expanded previous findings of observed hypo-connectivity within the salience network itself and between the SN and DMN in ASD^18,53^. The aberrant DMN-SN interaction might potentially be the origin of deficits seen in ASD regarding impaired emotional awareness of the self and others, and impaired reorienting to salient social or emotional stimuli.

Finally, factor 3 showed negative coupling relations among the DMN and between DAN nodes. In particular, the posterior subregion of the right TPJ depicted lower functional coupling than the anterior subregion, while no such dissociation was observed in the left TPJ. In contrast, factor 2 showed the inverse coupling pattern, while overall showing more positive associations with ASD than ADHD. Earlier studies found a functional separation of the anterior and posterior rTPJ^37,54^: While the anterior subregion was shown to be closely related to the reorientation of attention, the posterior cluster was functionally associated with Theory-of-Mind and social cognition. Across brain phenotypes, distinct patterns of dysconnectivity in the rTPJ effectively differentiated between ADHD and ASD. We hence suggest that a shared expression of factors 2 and 3 may play a critical role in contributing to the variability of shared deficits seen in both disorders.

Connectivity-derived biomarkers anchored in the partly shared functional architecture of the DMN may further disentangle the observed heterogeneity in ADHD and ASD diagnostics and potentially lead to targeted treatment options in the future. In ADHD, Peterson and colleagues specifically reported that psychostimulants may improve ADHD related symptoms by normalizing dysfunctional connections between DMN and DAN related activity in adolescents^55^. ASD, in turn, was reported to show aberrant intra-DMN coupling and diminished antagonistic correlation with task-positive networks, such as DAN and SN^56,57^. However, dedicated translational research will be needed to extend the search for transdiagnostic biomarkers and eventually evaluate their potential use in treatment.

In conclusion, we used an innovative hierarchical Bayesian modeling strategy to identify and formalize intermediate brain phenotypes to interrogate our hypothesis of shared dysfunctional connectivity in the DMN, DAN, and SN. The endo-phenotypes derived in a data-driven fashion without access to any clinical or diagnostic information were gradually shared across the neurodevelopmental disorders of ADHD and ASD. We demonstrated that hundreds of resting-state brain scans for each participant could be re-expressed in only three numbers that captured hidden heterogeneity in DMN coupling. The derived brain endo-phenotypes were then demonstrated to enhance categorical diagnoses made by board-certified psychiatrists to capture the neural dysfunction shared in both disorders more accurately. The realized analysis strategy is not constrained to ADHD and ASD, but may be applied to a variety of major psychiatric disorders. Further investigations may target not only shared dysfunction^58^ but also individual treatment response, similar to recent work in depression^59^. Identifying and validating brain-based endo-phenotypes will most likely be and continue to be an unavoidable cornerstone for personalized medicine in child psychiatry^26,60^ and general psychiatry^26,27,61^.

### Data availability

All used data are open-access (ABIDE and ADHD-200) and are readily accessible to the reader.
